# Te-rP-C Anodes Prepared Using a Scalable Milling Process for High-Performance Lithium-Ion Batteries

**DOI:** 10.3390/mi14122156

**Published:** 2023-11-25

**Authors:** Woo Seok Choi, Minseo Kim, Il Tae Kim

**Affiliations:** Department of Chemical and Biological Engineering, Gachon University, Seongnam-si 13120, Republic of Korea; polonium04@naver.com (W.S.C.); minxissh@gachon.ac.kr (M.K.)

**Keywords:** tellurium, red phosphorus, carbon matrix, high-energy ball milling, high-rate capability, Li-ion battery anodes

## Abstract

Red phosphorus (rP) is one of the most promising anode materials for lithium-ion batteries, owing to its high theoretical capacity. However, its low electronic conductivity and large volume expansion during cycling limit its practical applications, as it exhibits low electrochemical activity and unstable cyclability. To address these problems, tellurium (Te)-rP-C composites, which have active materials (Te, rP) that are uniformly distributed within the carbon matrix, were fabricated through a simple high-energy ball milling method. Among the three electrodes, the Te-rP (1:2)-C electrode with a 5% FEC additive delivers a high initial CE of 80% and a high reversible capacity of 734 mAh g^−1^ after 300 cycles at a current density of 100 mA g^−1^. Additionally, it exhibits a high-rate capacity of 580 mAh g^−1^ at a high current density of 10,000 mA g^−1^. Moreover, a comparison of the electrolytes with and without the 5% FEC additive demonstrated improved cycling stability when the FEC additive was used. Ex situ XRD analysis demonstrated the lithiation/delithiation mechanism of Te-rP (1:2)-C after cycling based on the cyclic voltammetry results. Based on the electrochemical impedance spectroscopy analysis results, a Te-rP-C composite with its notable electrochemical performance as an anode can sufficiently contribute to the battery anode industry.

## 1. Introduction

Rechargeable lithium-ion batteries (LIBs) have been widely utilized as energy storage systems for portable equipment such as laptops and cellphones, owing to their high operating voltage and low memory effects [1,2]. Currently, many electrode materials for cathodes and anodes have been studied to achieve better LIB performance. In the case of cathode materials, three main types of cathode materials with layered, spinel, and olivine structures have been developed. For eV vehicle applications, high Ni-based cathodes or LiFePO_4_ cathodes have been rigorously evaluated [3,4,5]. Currently, the commercially available anode material for LIBs is graphite, which has a low theoretical capacity of 372 mAh g^−1^. To improve the limited capacity of anodes, Si-based anode materials, including SiO_x_ [6,7], Si alloys [8,9], and Si/C nanocomposites [10], have also been evaluated [11,12,13]. These studies have attempted to make further improvements in the energy density, cyclability, and rate capability.

Lithium-alloyed materials have been intensively studied as potential anodes for LIBs because each atom can react electrochemically with various Li atoms. They typically have high theoretical capacities, and common examples include Li_4.4_Si (4200 mAh g^−1^) [14,15,16,17], Li_3_P (2596 mAh g^−1^) [18,19,20], Li_4.4_Sn (990 mAh g^−1^), and so on [21,22,23]. However, a major issue related to Li-alloying materials is the extreme volume expansion that occurs after Li^+^ insertion/extraction. The volume expansion results in the cracking and crumbling of the active materials, which leads to a rapid loss in capacity within a few cycles. One effective method to minimize the volume change is to introduce alloying active materials into the carbon matrix [24].

Recently, tellurium (Te)-based composites have been reported as potential anode materials for LIBs [25,26,27,28,29,30,31]. The electronic conductivity of tellurium is the highest (2.0 × 10^2^ S m^−1^) among nonmetallic elements, resulting in better electrochemical kinetics [32]. Tellurium has a lower theoretical specific capacity of 420 mAh g^−1^ and electrochemically alloys with Li to form Li_2_Te. Additionally, tellurium has a high theoretical volumetric capacity of 2621 mAh cm^−3^ owing to its high material density of 6.24 g cm^−3^. Despite the several advantages of Te as a promising material, very few Te-based composites have been used as anode materials in LIBs.

Wang et al. were the first to prepare a tellurium/porous carbon (Te/C) composite using a vacuum-liquid-infusion method [29]. The Te/C electrode delivered an initial reversible capacity of 300 mAh g^−1^ at a current rate of 50 mA g^−1^ between 0.8 and 2.5 V, and still retained 87% of its initial capacity over 1000 cycles. Using a hydrothermal method, Guo et al. developed a tellurium@microporous carbon composite (Te@MPC), which exhibited a reversible capacity of 372 mAh g^−1^ over 280 cycles with a capacity retention of 90% at 42 mA g^−1^ between 1.0 and 2.7 V [25]. However, these energy densities are still insufficient for practical use. The red phosphorus (rP) could be used as one of the most promising materials because of its high theoretical capacity of 2596 mAh g^−1^, which is seven times more than that of commercial graphite. Furthermore, among its allotropes, rP is a chemically stable, cheap, easy-to-handle, and non-toxic material (red P, black P, and white P). Specifically, crystalline rP has critical disadvantages, including its huge volume expansion (>490%) during the Li^+^ insertion/extraction process, poor electronic conductivity (1.0 × 10^−14^ S cm^−1^), sluggish electrochemical kinetics, and irreversible storage capacity [20,33,34,35]. These would lead to poor electrochemical performance of the crystalline rP with the pulverization of the electrodes, poor rate performance, and minimal reversible capacity. One of the most successful methods for accommodating the volume variations during cycling is the application of amorphous rP. Amorphous rP lacks a well-defined crystal structure, and has characteristics including high surface area and fast electrochemical kinetics that can accommodate the strain associated with lithiation/delithiation, leading to better cycling stability [36,37,38,39]. Therefore, it is necessary to develop suitable composite systems to understand the two aspects of high-energy density and the mitigation of vulnerable volume changes during cycling [40].

In this study, to address the problems encountered when Te or rP are used independently, we fabricated a Te-rP-C nanocomposite using high-energy ball milling (HEBM), in which carbon was utilized to minimize volume expansion and improve the electrical conductivity of the composite system. The physical properties of the as-prepared Te-rP-C nanocomposite were thoroughly characterized, and it exhibited outstanding cyclability and high-rate capability as an LIB anode. Additionally, the lithiation/delithiation mechanism of the Te-rP-C nanocomposite electrode was evaluated using ex situ X-ray diffraction (XRD) based on cyclic voltammetry (CV) results. Owing to the simple and well-distributed composite morphology of the Te-rP-C materials, the Te-rP (1:2)-C electrode exhibited stable electrochemical performance, indicating its potential for use in next-generation LIB anodes.

## 2. Experiment

### 2.1. Synthesis of Te-rP-C Powder

The Te-rP-C powders were synthesized using tellurium (−200 mesh, 99.8%, Aldrich, China), commercial red phosphorus (−100 mesh, red amorphous, 98.9%, Alfa Aesar, Germany), and acetylene black (100% compressed, 99.9%, Alfa Aesar, United States) as the raw materials. The atomic ratios of Te to phosphorus were 2:1, 1:1, and 1:2, respectively. Subsequently, the Te-rP-C and acetylene black mixture at a ratio of 7:3 were mixed in a well. The raw materials were placed in a zirconia bowl (80 cm^3^) with ZrO_2_ balls (0.5 and 0.25 inch diameters). HEBM (Pulverisette 5 Planetary Mill, Fritsch GmbH, Germany) was performed at 300 rpm in an Ar atmosphere for 24 h.

### 2.2. Materials Characterization

The crystal structures of the Te-rP (1:1), Te-rP (2:1)-C, Te-rP (1:1)-C, and Te-rP (1:2)-C samples were determined using X-ray diffraction (XRD, Rigaku 2200, Japan) at an operating scan rate of 2° min^−1^ over 20–50° as well as transmission electron microscopy (TEM, JEOL JEM2100, Japan) with energy-dispersive X-ray spectroscopy (EDX, equipped with the HRTEM). The structural changes during cycling were analyzed using ex situ XRD and scanning electron microscopy (SEM, Hitachi S-4700, Japan).

### 2.3. Electrochemical Measurements

To fabricate the electrodes as anodes, the as-prepared powders were cast with polyvinylidene fluoride (PVDF, Aldrich, 12 wt.% in NMP) as a binder and Super P as a conducting agent at a mass ratio of 70:15:15 onto a Cu substrate, followed by drying at 70 °C in a vacuum oven. The dried electrode was punched into a 12 mm disk shape, and the loading of the active material was about 0.85–1.00 mg cm^−2^. Then, the electrodes were assembled into CR2032-type coin cells in a glovebox using a polyethylene separator and lithium foil as the counter electrodes. A 1 M solution of LiPF_6_ (ethylene carbonate (EC): diethyl carbonate (DEC) = 1:1, volume ratio) was used as the electrolyte (~120 μL), with and without an additive (5 vol.% FEC). The discharge/charge tests were conducted in the voltage range of 0.01–2.5 V (Li vs. Li^+^) at a current density of 0.1 A g^−1^ using the WBCS3000 battery cycler (WonAtech, Seoul, Korea) at 25 °C. Electrochemical impedance spectroscopy (EIS) and cyclic voltammetry (CV) measurements were performed using a ZIVE MP 1 system (WonAtech, Seoul, Korea). The EIS tests were conducted after 100 cycles at 100 kHz and 100 mHz. CV was performed in the range of 0.001–2.5 V (Li vs. Li^+^) at a scanning rate of 0.1 mV s^−1^.

## 3. Results and Discussion

**Figure 1:**
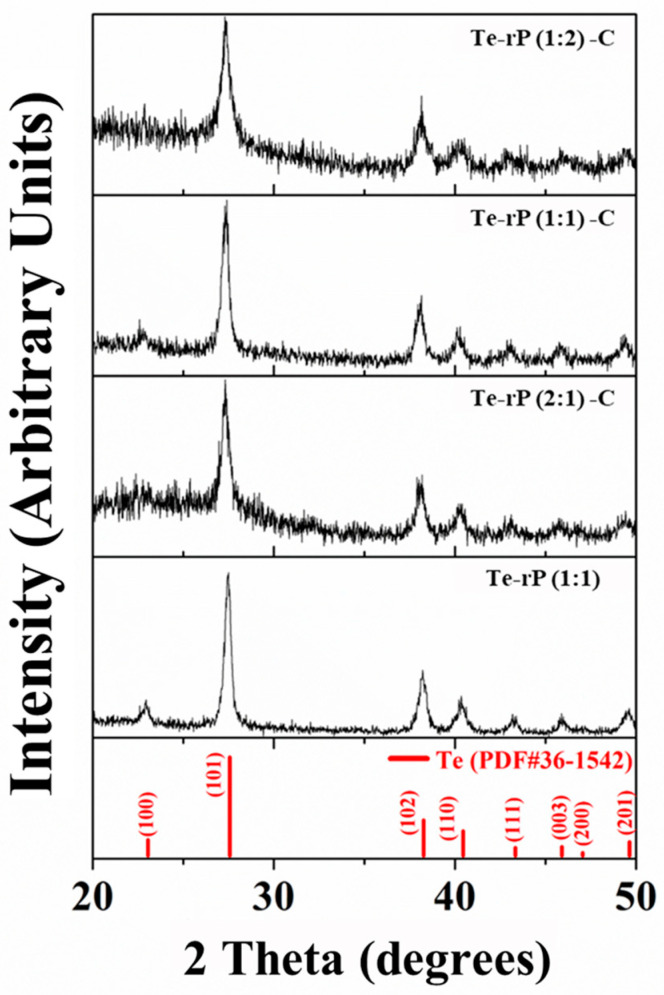
XRD patterns of Te-rP (1:1), Te-rP (2:1)-C, Te-rP (1:1)-C, and Te-rP (1:2)-C composite materials.

Powder X-ray diffraction (XRD) was used to evaluate the phase structures of the Te-rP-C composites. Figure 1 shows the XRD patterns of the Te-rP (1:1), Te-rP (2:1)-C, Te-rP (1:1)-C, and Te-rP (1:2)-C composites. The XRD pattern of rP is also presented in Figure S1, which indicates that rP is amorphous. All of the diffraction patterns of the prepared powder corresponded to a hexagonal Te phase (PDF#36-1452). There were no other crystalline phases because the rP and carbon were amorphous. Note that C and rP are amorphous. Therefore, this seems to broaden the XRD peaks in the Te-rP-C composite materials [30]. Therefore, it can be inferred that the Te-rP-C composites consist of Te and rP distributed in an amorphous carbon matrix.

**Figure 2:**
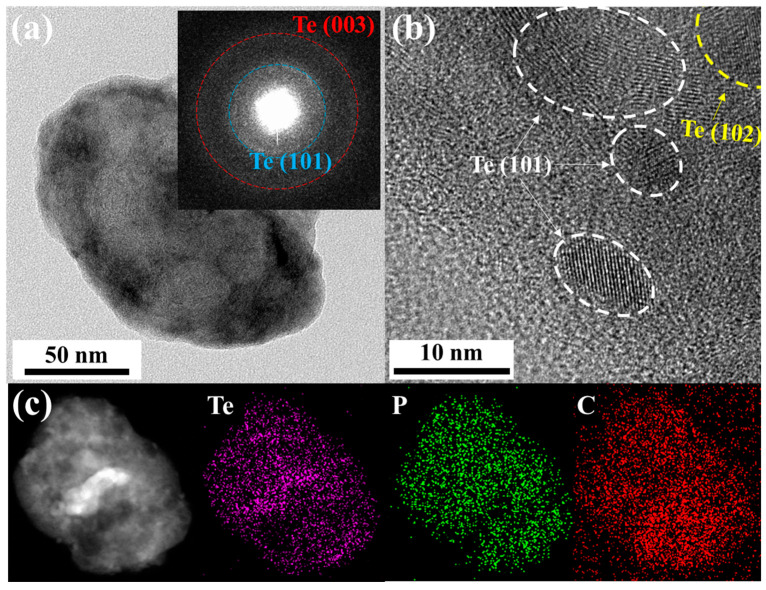
(**a**) TEM image of Te-rP (1:2)-C composite; inset shows the selected area electron diffraction pattern. (**b**) High-resolution TEM image of Te-rP (1:2)-C composite. (**c**) Low-magnification TEM image and corresponding elemental mapping images of Te-rP (1:2)-C composite.

Transmission electron microscopy (TEM) with energy-dispersive X-ray spectroscopy (EDX) was used to characterize the morphology of the obtained Te-rP-C material. Figure 2a shows a low-magnification TEM image of the Te-rP (1:2)-C composite. The inset of Figure 2a shows the selected-area electron diffraction pattern. Two ring patterns corresponding to the (101) and (003) planes of the Te crystal structure were observed. To further evaluate the specific morphology of Te-rP-C, an HRTEM image was obtained, as shown in Figure 2b. It shows the crystalline nanoparticles (5–15 nm in diameter) embedded in the amorphous carbon matrix, which show two d-spacings of 0.32 nm and 0.23 nm, corresponding to the (101) and (102) planes for the tellurium phase, respectively. The elemental mapping images shown in Figure 2c suggest a uniform distribution of Te and phosphorus in the amorphous carbon matrix. Thus, as discussed earlier, it can be concluded that crystalline Te and amorphous rP are well dispersed in the composite system. The TEM images of the other Te-rP-C materials synthesized at various ratios, in addition to Te-rP (1:2)-C, are summarized in Figure S2; the morphological characteristics are similar.

**Figure 3:**
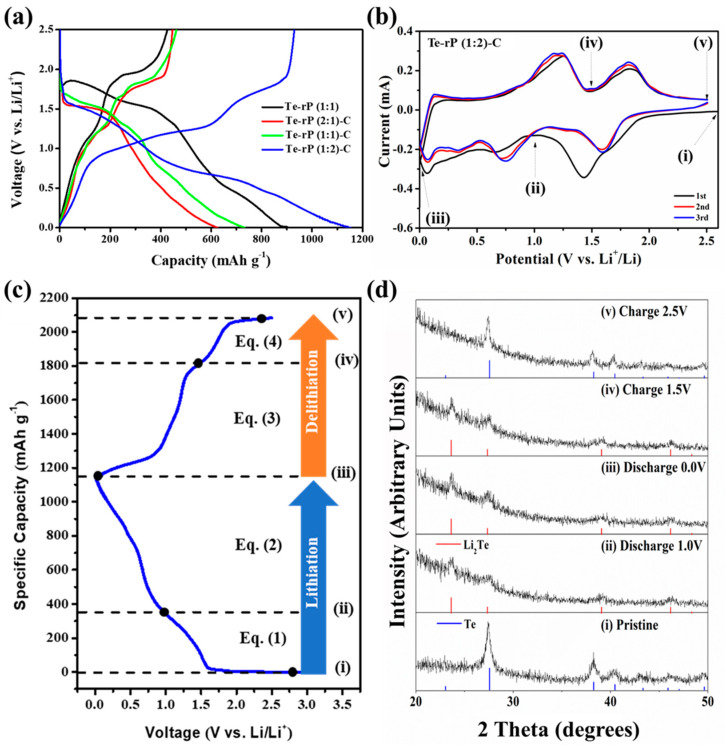
(**a**) Initial voltage profiles of the Te-rP (1:1), Te-rP (2:1)-C, Te-rP (1:1)-C, and Te-rP (1:2)-C at 100 mA g^−1^ with 5% FEC. (**b**) Cyclic voltammetry of Te-rP (1:2)-C anode at 0.1 mV s^−1^. (**c**) Voltage profile of Te-rP (1:2)-C anode and suggested reaction mechanisms. (**d**) Ex situ XRD patterns of Te-rP (1:2)-C electrode: (i) pristine, (ii) after discharge to 1.0 V, (iii) after full discharge to 0.0 V, (iv) after charging to 1.5 V, and (v) after full charging to 2.5 V.

Figure 3a shows the galvanostatic discharge and charge behaviors of the Te-rP (1:1), Te-rP (2:1)-C, Te-rP (1:1)-C, and Te-rP (1:2)-C electrodes with 5% FEC at 100 mA g^−1^ in the voltage ranges between 0.001 and 2.5 V (vs. Li/Li^+^). The galvanostatic discharge and charge behaviors of the electrodes without FEC are summarized in Figure S3. All of the electrodes exhibit a low Coulombic efficiency (CE) in the first cycle, which is attributed to the formation of the SEI film and the reduction in electrolyte decomposition (e.g., EC, PC, and FEC) on the electrode surface. However, as will be shown later, all of the electrodes exhibited an increased CE of over 90% from the 3^rd^ cycle, and demonstrated diminishing irreversible reactions by the SEI film. The initial charge capacity and initial Coulombic efficiency (ICE) of the Te-rP (1:1) electrode are 426 mAh g^−1^ and 47%, respectively. This low ICE was due to the large number of side reactions on the surface of the electrode resulting from the absence of carbon during cycling. The Te-rP (1:1)-C electrode has an initial charge capacity of 462 mAh g^−1^ with an ICE of 63%; this ICE value is higher than that of the Te-rP (1:1) electrode because the carbon matrix plays a role in alleviating the volume change as well as reducing vigorous side reactions, establishing metastable SEI layer formation. Meanwhile, the Te-rP (2:1)-C and Te-rP (1:2)-C electrodes exhibit initial charge capacities of 447 and 929 mAh g^−1^, respectively, which correspond to ICEs of 72 and 80%, respectively. As expected, the Te-rP (1:2)-C electrode exhibits the highest initial discharge capacity. The lower capacities of Te-rP (2:1)-C and Te-rP (1:1)-C are due to the lower amounts of phosphorus in the two samples.

To understand the lithiation/delithiation mechanism, CV curves of the initial three scans were analyzed with a scan rate of 0.1 mV s^−1^ within 0.001–2.5 V. Figure 3b shows the CV curves of the Te-rP (1:2)-C electrode, and Figure S4 presents the CV profiles of the Te-rP (2:1)-C, Te-rP (1:1)-C, and Te-rP (1:1) electrodes. As shown in Figure 3b, a broad peak at 0.7 V was observed during the first discharge scan, which corresponds to the reduction/decomposition of the electrolytes to form SEI films on the surface of the electrodes. A peak was also observed between 1.50 V and 1.75 V in the discharge scan, which can be attributed to the reaction of lithium ions and tellurium. When the potential was further scanned from 1.0 to 0.5 V, a peak near 0.75 V appeared, corresponding to the reaction between lithium ions and phosphorus. It is noted that the as-prepared electrodes showed different initial voltage profiles based on the ratio of Te and rP. The Te-rP (1:1) electrode reveals a very blurry initial voltage profile due to the unstable electrochemical reaction. The Te-rP (1:1)-C electrode shows a distinct two-step electrochemical reaction with similar current intensity; the redox reaction occurring at 1.5/1.75 V corresponds to the reaction between Li ions and Te, while the redox reaction occurring at 0.5/1.0 V represents the reaction between Li ions and rP. The Te-rP (2:1)-C electrode exhibits a larger current intensity at the redox reaction occurring at 1.5/2.0 V, while the Te-rP (1:2)-C electrode shows larger current intensity at the redox reaction occurring at 0.5/1.0 V. This could be due to the different amounts of Te and rP in the composites. In the subsequent charge scan, two overlapped anodic peaks are centered at 1.2 V and 1.8 V, which should correspond to lithium ion extraction from the charged phases. The overlapping CV curves in the subsequent cycles indicate the superior electrochemical performance of the Te-rP (1:2)-C electrode.

To further confirm the lithiation/delithiation mechanism of Te-rP (1:2)-C, ex situ XRD (Figure 3d) analysis was conducted at different cycling states based on the CV (Figure 3b), and Figure 3c shows the corresponding voltage profile. When the electrode was discharged to 1.0 V (Figure 3d(ii)), the Te hexagonal phase was completely transformed into the Li_2_Te cubic phase (PDF#23-0370). When further discharged to 0.001 V (Figure 3d(ii,iii)), there seemed to be no additional phase change, which could be due to the formation of amorphous Li_3_P phases during discharge. Moreover, when the electrode was charged to 1.5 V, the ex situ XRD patterns remained, which could be due to the formation of amorphous rP [41]. When fully charged to 2.5 V (Figure 3d(v)), all of the XRD peaks aligned with those of the Te phases. Therefore, the ex situ XRD results confirmed the reversible lithiation/delithiation of the Te-rP (1:2)-C electrode. Based on the CV and ex situ XRD analyses, the electrochemical reaction mechanisms can be summarized as follows:

(1) $$Te+2{Li}^{+}+2e^{-}⟶{Li}_{2}Te\;\;\left(\text{Discharge}\;\text{at}\;1.0\;V\right)$$

(2) $$(\mathrm{amorphous})\;P+3{Li}^{+}+3e^{-}⟶{Li}_{3}P\;\;\left(\text{Discharge}\;\text{at}\;0.001\;V\right)$$

(3) $$(\mathrm{amorphous})\;{Li}_{3}P⟶P+3{Li}^{+}+3e^{-}\;\;\left(\text{Charge}\;\text{at}\;1.5\;V\right)$$

(4) $${Li}_{2}Te⟶Te+2{Li}^{+}+2e^{-}\;\left(\text{Charge}\;\text{at}\;2.5\;V\right)$$

**Figure 4:**
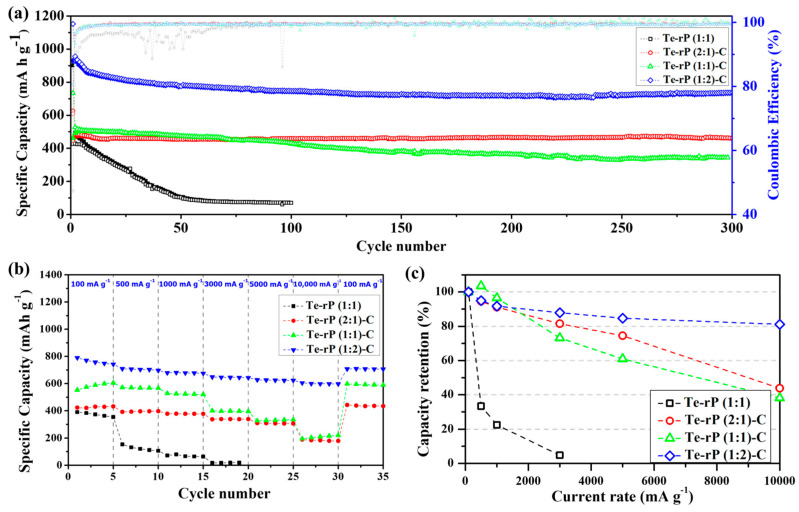
(**a**) Cycling performances of the Te-rP (1:1), Te-rP (2:1)-C, Te-rP (1:1)-C, and Te-rP (1:2)-C with 5% FEC additive. (**b**) Rate cyclability and (**c**) normalized capacity retention values (percent) of the as-prepared electrodes with 5% FEC additive.

The as-prepared Te-rP-C electrodes were used for the LIB anodes, as shown in Figure 4. Figure 4a shows the cycling performance of Te-rP (1:1), Te-rP (2:1)-C, Te-rP (1:1)-C, and Te-rP (1:2)-C with a 5% FEC additive in the electrolyte. In the case of Coulombic efficiency (CE) of the electrodes, the initial Coulombic efficiencies (ICE) are 47%, 72%, 63%, and 80% for Te-rP (1:1), Te-rP (2:1)-C, Te-rP (1:1)-C, and Te-rP (1:2)-C, respectively (Figure 4a). The Te-rP (1:1) electrode without C reveals a very low and fluctuating CE, which could be caused by unstable electrochemical reactions. On the other hand, the Te-rP-C electrodes show a very stable CE; the CE rapidly reaches approximately 98% and approaches 100% during the prolonged cycles. For the Te-rP (1:1) electrode, the capacity decreased significantly within 50 cycles because severe volume changes occurred during the lithiation/delithiation process. To alleviate the volume expansion, a carbon matrix should be introduced into the alloy systems [24,42,43,44]. The capacity retention of the Te-rP (1:1)-C electrode was significantly better than that of the Te-rP (1:1) electrode. The Te-rP (1:1)-C electrode delivered a charge capacity of 344 mAh g^−1^ at 300 cycles and 68% of the charge capacity with stable cycling. Furthermore, when the molar ratios of tellurium to phosphorus were 2:1 and 1:2, the charge capacities of the Te-rP (2:1)-C and Te-rP (1:2)-C composite electrodes also exhibited very stable cyclability with capacities of 460 mAh g^−1^ at 96% and 734 mAh g^−1^ at 79% of the capacity retention after 300 cycles, respectively. It is noted that at the 200th cycle, the reversible capacity of the Te-rP (1:2)-C composite electrode is 720 mAh g^−1^, and that at the 300th cycle is 739 mAh g^−1^. It shows somewhat enhanced capacity value. This may be due to the formation of the additional electrochemical reactions caused by polymeric gel-like film (electrochemical decomposition of electrolyte) on the surface of the electrode materials [45]. As mentioned earlier, the theoretical capacity of phosphorus was 2596 mAh g^−1^, assuming the formation of the Li_3_P phase. Therefore, for the Te-rP (1:2)-C electrode, a large amount of phosphorus led to an increase in capacity.

Furthermore, the enhancement of the cycling properties could be due to the well-dispersed active Te nanoparticles with high electronic conductivity, as well as the rP and coverage of the highly conductive carbon matrix. The existence of the carbon matrix mitigated the large volume expansion, leading to better cyclability (refer to the results for the Te-rP (1:1) and Te-rP-C (1:1) electrodes). The incorporation of an appropriate amount of the FEC additive also served as a positive factor to form a stable SEI layer. Based on these positive characteristics, the Te-rP-C electrodes exhibited significantly improved cycling performance. However, the capacity retention of the electrodes without the FEC additive was inferior to that of the electrodes with the FEC additive, as shown in Figure S5a. The Te-rP (1:1) electrode exhibited very poor cycling properties. The Te-rP (1:1)-C electrode demonstrated a significant decrease in capacity when FEC was not added, providing a charge capacity of 346 mAh g^−1^ at 80 cycles with 66%. The Te-rP (2:1)-C and Te-rP (1:2)-C electrodes at 300 cycles were 360 mAh g^−1^ and 539 mAh g^−1^, respectively, corresponding to capacity retentions of 77% and 67%. This characteristic may arise from the synergistic effect generated from the optimal ratio of Te and rP in the conductive matrix. To confirm this phenomenon, further research will be performed, including further changing the ratio between active materials and applying appropriate simulations to check the interactions between the materials. These values are lower than those of the Te-rP (1:2)-C and Te-rP (2:1)-C electrodes with the FEC additive because they help to form thin and stable SEI films and reduce the amount of electrolyte decomposition. Therefore, it can be concluded that the FEC additive significantly affects the electrochemical properties.

Moreover, to evaluate the fast recharging property, the rate capability and capacity retention of the as-obtained electrodes with the FEC additive were tested, as shown in Figure 4b,c. The test was conducted at 0.001–2.5 V (vs. Li/Li^+^) at various current densities. The Te-rP (1:1) electrode presented inferior rate capability and a low charge capacity of 15 mAh g^−1^ at 3000 mA g^−1^, corresponding to a very poor capacity retention of 5% when normalized by the capacities at 100 mA g^−1^. However, the presence of a carbon matrix leads to better rate capability. For instance, the Te-rP (1:1)-C and Te-rP (2:1)-C electrodes demonstrated better rate performances than the Te-rP (1:1) without a carbon matrix (Figure 4c,d). The rate performance of the Te-rP (1:2)-C electrode was the best. The Te-rP (1:2)-C electrode exhibited charge capacities of 698, 675, 647, and 623 mAh g^−1^ at current densities of 500, 1000, 3000, and 5000 mA g^−1^, respectively, corresponding to capacity retentions of 95, 92, 88, and 85%, respectively. At a very high current density of 10,000 mA g^−1^, the Te-rP (1:2)-C electrode showed an outstanding capacity retention of 81%. These notable differences in the rate properties could be due to the optimized Te-rP-based composite electrodes, where the formation of well-dispersed Te with rP at an optimum ratio in the conductive carbon matrix could lead to a synergistic effect for achieving high-performance Li-ion cells. The electrodes with the FEC additive exhibited better performance than the electrodes without FEC. For instance, in the case of Te-rP (1:1)-C (Figure S5), the capacity retention of the electrode without FEC was 4% at 10,000 mA g^−1^, whereas that of the electrode with FEC was 38% at 10,000 mA g^−1^. These superior rate capabilities can also be attributed to the formation of thin and stable SEI films, which reduce the number of Li^+^ diffusion paths.

**Figure 5:**
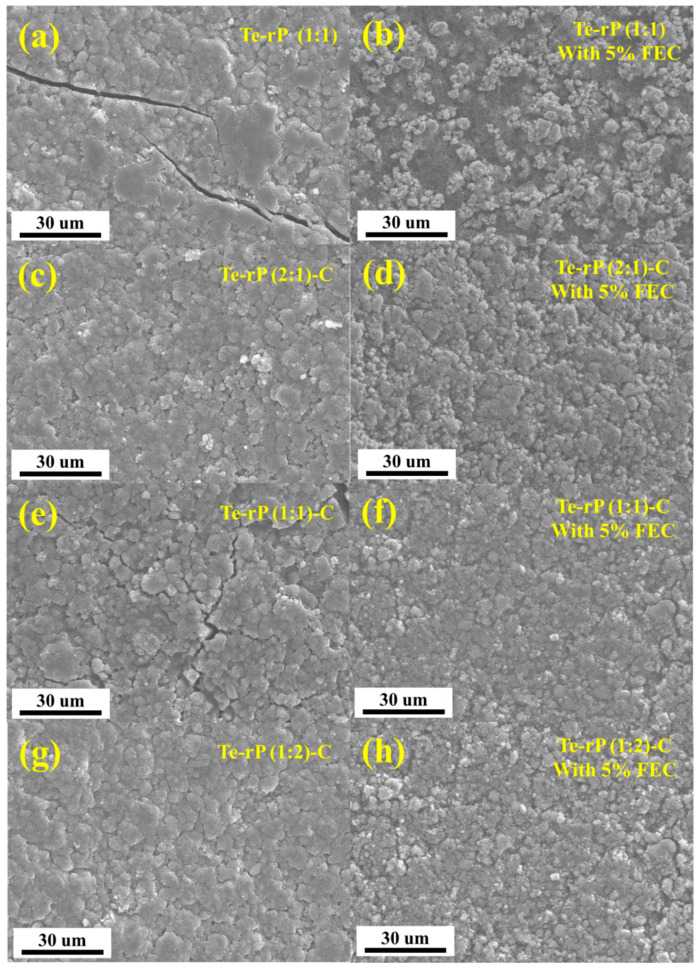
SEM images of electrodes: (**a**) Te-rP, (**b**) Te-rP with 5% FEC, (**c**) Te-rP (2:1)-C, (**d**) Te-rP (2:1)-C with 5% FEC, (**e**) Te-rP (1:1)-C, (**f**) Te-rP (1:1)-C with 5% FEC, (**g**) Te-rP (1:2)-C, and (**h**) Te-rP (1:2)-C with 5% FEC after 100 cycles.

After cycling, checking the morphology of the as-prepared electrodes via ex situ SEM analysis is essential. After 100 cycles, the cells were opened in an Ar-filled glove box, rinsed with diethylene carbonate, dried, and their morphologies were observed (Figure 5). In the case of the Te-rP (1:1) electrode without the FEC, numerous prominent cracks were observed (Figure 5a), whereas the Te-rP (1:1) electrode with FEC did not exhibit many cracks; however, particle agglomeration was observed (Figure 5b). In contrast, the other electrodes forming a carbon matrix exhibited narrower and fewer cracks, demonstrating the inhibition of volume expansion during cycling. Notably, the Te-rP (1:2)-C and Te-rP (2:1)-C electrodes without the FEC additive did not exhibit many cracks, leading to better electrochemical properties than those of the Te-rP (1:1) and Te-rP (1:1)-C electrodes without FEC. Nevertheless, their reversible capacities were lower than those of the Te-rP (1:2)-C and Te-rP (2:1)-C electrodes containing FEC, which formed thin and stable SEI layers. In the SEM images of the Te-rP (1:2)-C and Te-rP (2:1)-C electrodes with and without the FEC, the electrodes without the FEC did not exhibit the morphology consisting of each nanoparticle, but revealed blurred and thick SEI layer formation. The results obtained from the SEM analysis were consistent with the overall electrochemical performance of the cells.

**Figure 6:**
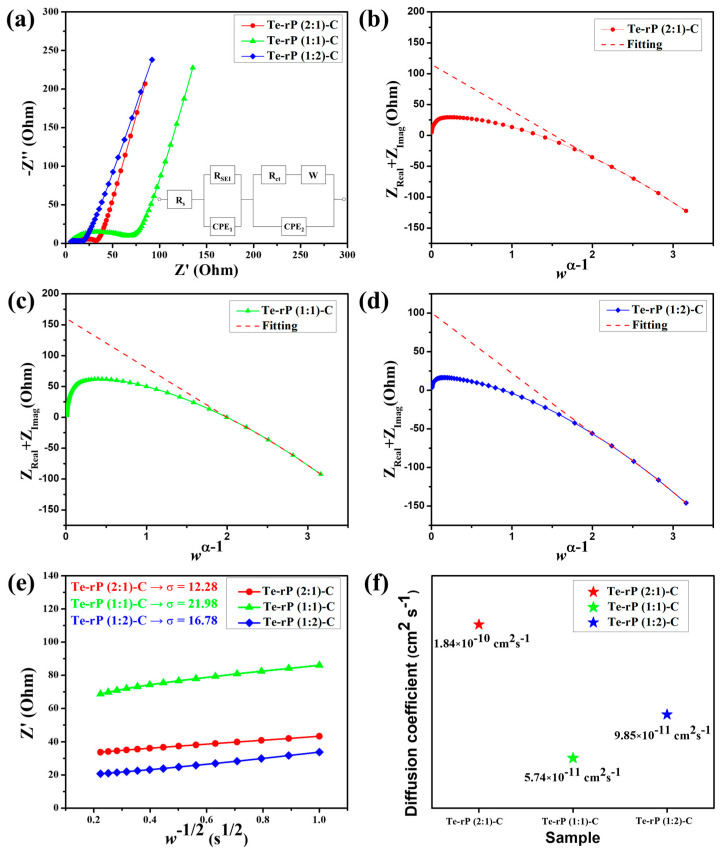
(**a**) EIS curves of the Te-rP (2:1)-C, Te-rP (1:1)-C, and Te-rP (1:2)-C electrodes and equivalent circuit models. (Z_Real_ + Z_Imag_) plots with respect to w^α−1^ of various electrodes after 50th cycle: (**b**) Te-rP (2:1)-C, (**c**) Te-rP (1:1)-C, and (**d**) Te-rP (1:2)-C. (**e**) Linear fitting plot of w^−1/2^ versus Z’ plot. (**f**) Calculated Li-ion diffusion coefficients for electrodes with the 5% FEC additive.

The superior cycling and rate performances were further characterized using electrochemical impedance spectroscopy (EIS). The cells used for EIS analysis were run for 50 cycles at a current density of 500 mA g^−1^. The tests were conducted in a fully charged state (2.5 V (vs. Li/Li^+^)). As shown in Figure 6a, the Nyquist plots consist of a semicircle and a linear slope. The semicircle in the high–medium-frequency region corresponds to the SEI and charge transfer resistance, whereas the linear slope in the low-frequency region is related to the bulk resistivity [46,47]. The equivalent circuit included R_s_, R_SEI_, R_ct_, and W, which correspond to the surface resistance, SEI resistance, charge-transfer resistance, and Warburg element, respectively. Two constant-phase elements, denoted as CPE_1_ and CPE_2_, are used for capacitance contributions arising from the solid electrolyte interphase (SEI) layers and the active material, respectively [48]. Table S1 summarizes the obtained values of R_s_, R_SEI_, and R_ct_ with refinement by applying a low-frequency constraint [49]. According to Vo et al., the y-intercept in the (Z′ + Z″) versus the w^α−1^ plot is the sum of R_s_, R_ct_, and R_SEI_ (w and α are the frequency and fractal dimension of CPE_2_, respectively). Figure 6b shows a value of 114.96 Ω for the sum of R_s_ + R_SEI_ + R_ct_ for the Te-rP (2:1)-C composite electrode. The R_SEI_ of the Te-rP (2:1)-C electrode refined using CNLS is 83.28 Ω. Likewise, the sum of R_s_ + R_SEI_ + R_ct_ for the Te-rP (1:1)-C composite electrode is 160.40 Ω, and the R_SEI_ value is 86.29 Ω. The sum of R_s_ + R_SEI_ + R_ct_ for the Te-rP (1:2)-C composite electrode is 100.53 Ω, and the R_SEI_ value is 82.37 Ω. In summary, at high–medium frequencies, the semicircle diameter of the Te-rP (1:2)-C electrode is the smallest, even though the difference in the values is not significant, indicating that the Te-rP (1:2)-C electrode has a more stable SEI film and lower charge-transfer resistance than the Te-rP (2:1)-C and Te-rP (1:1)-C electrodes. 

Regarding Li-ion dynamics, the Li-ion diffusion coefficient can be calculated as follows:

(5) $$Z^{\prime }=R_{s}+R_{SEI}+R_{ct}+{\sigma w}^{-1/2}$$

where Z′ is the real part in the resistance, w is the frequency, and σ is the Warburg factor. To estimate σ, one can perform linear regression on the w^−1/2^ versus Z plot, utilizing the Z′ and w values obtained from the Nyquist plot’s Warburg impedance region. Then, σ is approximated as the gradient of the linear fit [50]. The as-approximated σ values of the Te-rP (2:1)-C, Te-rP (1:1)-C, and Te-rP (1:2)-C electrodes are 12.28, 21.98, and 16.78, respectively (Figure 6e). Using these σ values, the diffusion coefficients of the different electrodes were calculated as follows [51]

(6) $$D_{{Li}^{+}}=\frac{R^{2}T^{2}}{{2A}^{2}n^{4}F^{4}C^{2}\sigma^{2}}$$

In this context, A corresponds to the contact area, equivalent to the electrode surface area. F represents the Faraday constant, C signifies the concentration of Li-ions in the electrolyte, and n is a dimensionless parameter (n = 1). Additionally, T stands for absolute temperature, R denotes the gas constant, and D_Li_^+^ represents the Li-ion diffusion coefficient. Figure 6f shows the D_Li_^+^ values for the Te-rP (2:1)-C, Te-rP (1:1)-C, and Te-rP (1:2)-C electrodes. The D_Li_^+^ value of the Te-rP (1:2)-C anode was calculated as 9.85 × 10^−11^ cm^2^ s^−1^, which was lower than that of the Te-rP (2:1)-C (1.84 × 10^−10^ cm^2^ s^−1^) and higher than that of the Te-rP (1:1)-C (5.74 × 10^−11^ cm^2^ s^−1^). Based on the resistance and diffusivity results, the Te-rP (1:2)-C electrode demonstrates low resistance and moderate Li-ion diffusivity when compared to the other two electrodes. Even though the Te-rP (1:2)-C electrode does not exhibit the highest Li diffusivity, which may be due to the larger amount of rP in the composite, the Te-rP (1:2)-C electrode exhibits the highest capacity values with stable cyclability. This could be due to the synergistic effect resulting from the formation of well-dispersed Te and rP at the optimum ratio in the conductive carbon matrix. Finally, similar studies reported previously were compared to our Te-rP-C composite electrodes. The results indicate that the as-developed Te-rP-C composite electrodes, specifically the Te-rP (1:2)-C electrode, represent reasonable electrochemical performance, as shown in Table S2. Therefore, it is inferred that the as-prepared Te-rP (1:2)-C could be a viable anode material for high-performance Li-ion batteries.

## 4. Conclusions

Te-rP-C composites were fabricated using HEBM, from which active materials (Te, rP) uniformly distributed within the carbon matrix were obtained. Te, rP, and carbon were used to enhance the electrical conductivity, increase the capacity, and buffer the large volume expansion during cycling. Ex situ XRD analysis was conducted to evaluate the lithiation/delithiation mechanisms of the Te-rP (1:2)-C electrode upon cycling based on the CV results, where a crystalline Li_2_Te phase was detected. For the electrochemical characteristics, the Te-rP (2:1)-C and Te-rP (1:2)-C electrodes with 5% FEC additive delivered a high initial CE of 72 and 80% and reversible capacities of 460 and 734 mAh g^−1^ at a current density of 100 mA g^−1^, respectively. They also exhibited high-rate capacities of 208 and 580 mA h g^−1^, respectively, at a high current density of 10,000 mA g^−1^. The composite electrodes with the FEC exhibited better electrochemical performance than those without the FEC additive. Based on various in-depth analyses, these outstanding performances could be due to the optimized Te-rP-based composite electrodes, where the formation of well-dispersed Te with rP in an optimum ratio in the conductive carbon matrix led to a synergistic effect for achieving high-performance Li-ion cells. However, it is also necessary to achieve stable full cells with high energy densities. In this regard, a potential study to develop a high-energy-density full cell can be conducted by applying the optimized Te-rP-C electrode and modulating parameters including the N/P ratio and cut-off voltages, and by fabricating novel architectures [52]. Overall, it is expected that the Te-rP-C composite anodes with their excellent performance will contribute significantly to the battery industry.

## Data Availability

The data presented in this study are available on request from the corresponding author.

## Supplementary Materials

The following supporting information can be downloaded at: https://www.mdpi.com/article/10.3390/mi14122156/s1, Figure S1: XRD pattern of rP.; Figure S2: TEM images; insets show the SAED pattern, HRTEM image, and elemental mapping images of (**a**) Te-rP (1:2)-C, (**b**) Te-rP (1:1)-C, and (**c**) Te-rP (1:1) composite.; Figure S3: Initial voltage profiles of the Te-rP (1:1)-C, Te-rP (2:1)-C, Te-rP (1:1)-C, and Te-rP (1:2)-C at 100 mA g^−1^ without the FEC additive.; **Figure S4**: Cyclic voltammetry of (**a**) Te-rP (2:1)-C, (**b**) Te-rP (1:1)-C, and (**c**) Te-rP (1:1) electrodes at 0.1 mV s^−1^.; Figure S5: (**a**) Cycling performance of the Te-rP (1:1)-C, Te-rP (2:1)-C, Te-rP (1:1)-C, and Te-rP (1:2)-C without the 5% FEC additive. (**b**) Rate cyclability and (c) normalized capacity retention values (percent) of as-prepared electrodes without FEC additive.; Table S1: EIS data of the Te-rP (2:1)-C, Te-rP (1:1)-C, and Te-rP (1:2)-C electrodes.; Table S2: Comparison of the electrochemical properties of Te, rP-related composite anodes [30,53,54,55,56,57,58].
