# Supplementary material for: Te-rP-C Anodes Prepared Using a Scalable Milling Process for High-Performance Lithium-Ion Batteries
Source: Micromachines (Basel). 2023 Nov 25;14(12):2156. doi: 10.3390/mi14122156 (PMC10745479; doi:10.3390/mi14122156)
Supplement: Supplementary file 1 [file micromachines-14-02156-s001.zip › micromachines-2714318-supplementary.pdf]

micromachines

MDPI

Supplementary Materials: Te-rP-C Anodes Prepared Using a Scalable Milling Process for High-Performance Lithium-Ion Batteries

Woo Seok Choi, Minseo Kim and Il Tae Kim *

Department of Chemical and Biological Engineering, Gachon University, Seongnam-si 13120, Republic of Korea; polonium04@naver.com (W.S.C.); minxissh@gachon.ac.kr (M.K.)

* Correspondence: itkim@gachon.ac.kr; Tel.: +82-31-750-8835; Fax: +82-31-750-5363

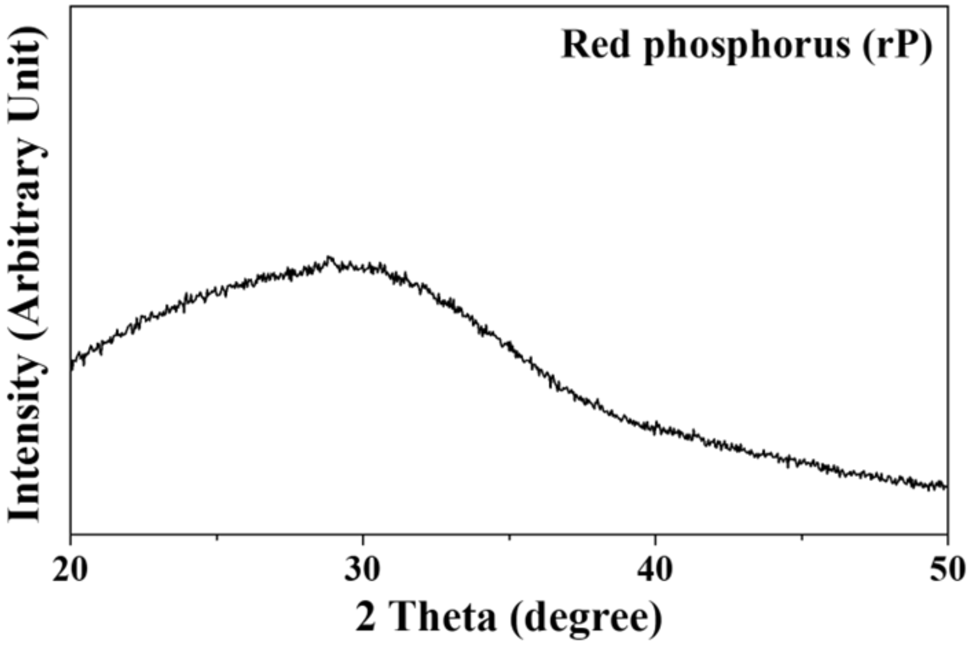

Figure S1. XRD pattern of rP.

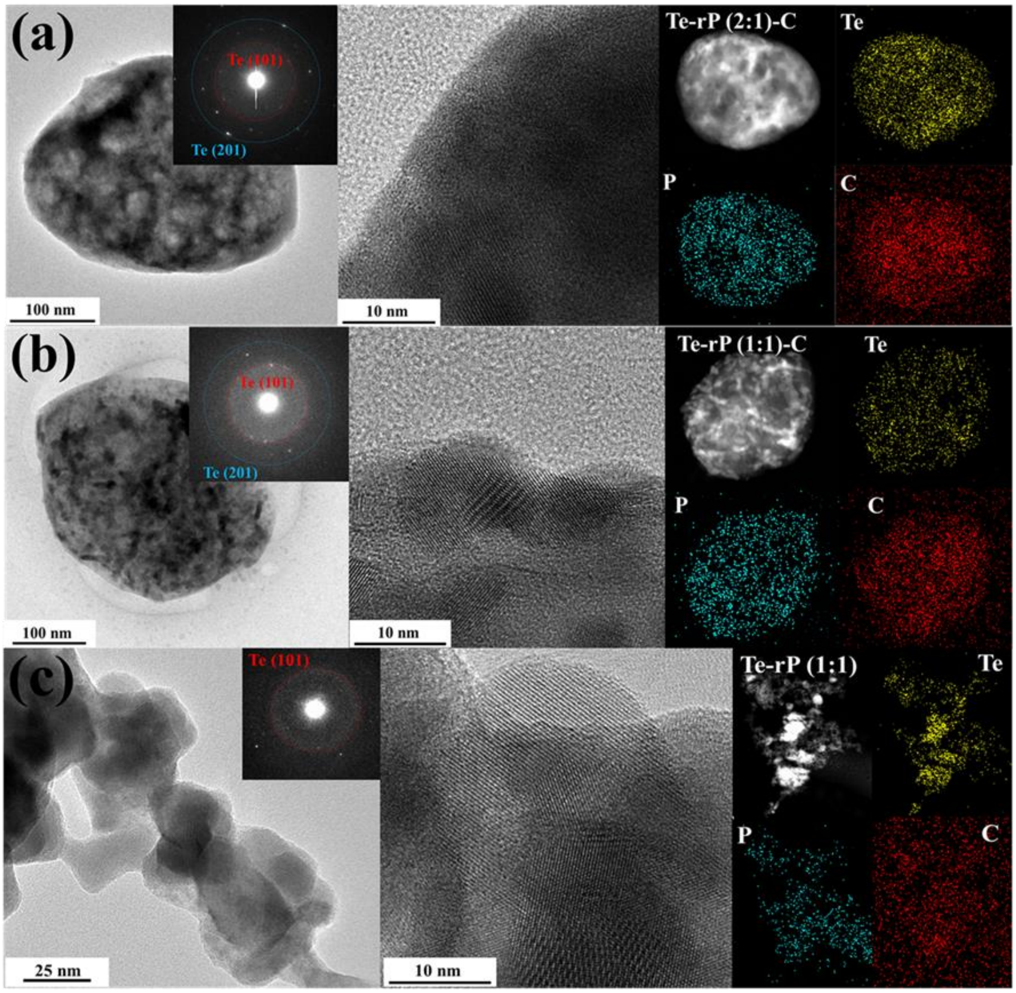

Figure S2. TEM images; insets show the SAED pattern, HRTEM image, and elemental mapping images of (**a**) Te-rP (1:2)-C, (**b**) Te-rP (1:1)-C, and (**c**) Te-rP (1:1) composites.

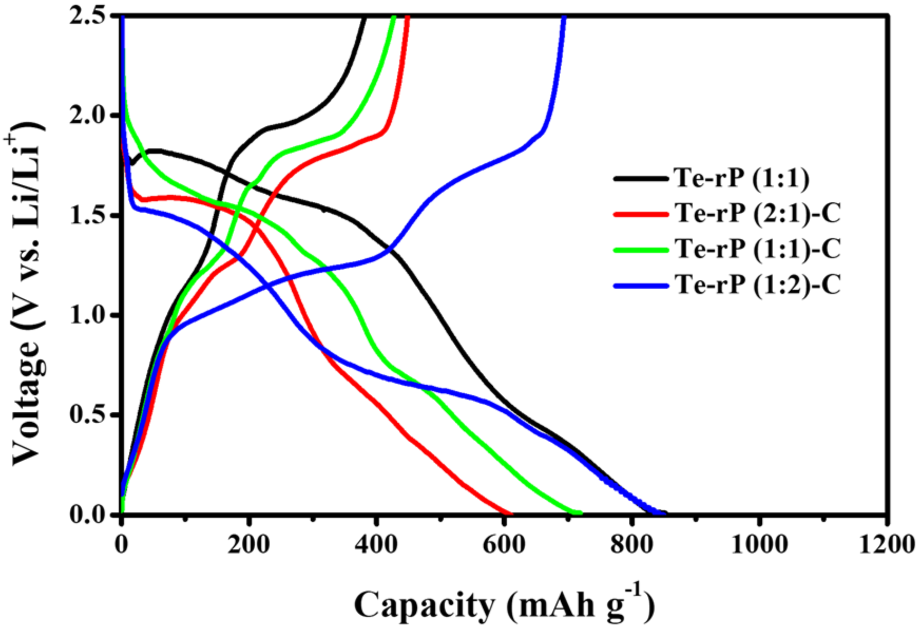

Figure S3. Initial voltage profiles of the Te-rP (1:1)-C, Te-rP (2:1)-C, Te-rP (1:1)-C, and Te-rP (1:2)-C at 100 mA g⁻¹ without the FEC additive.

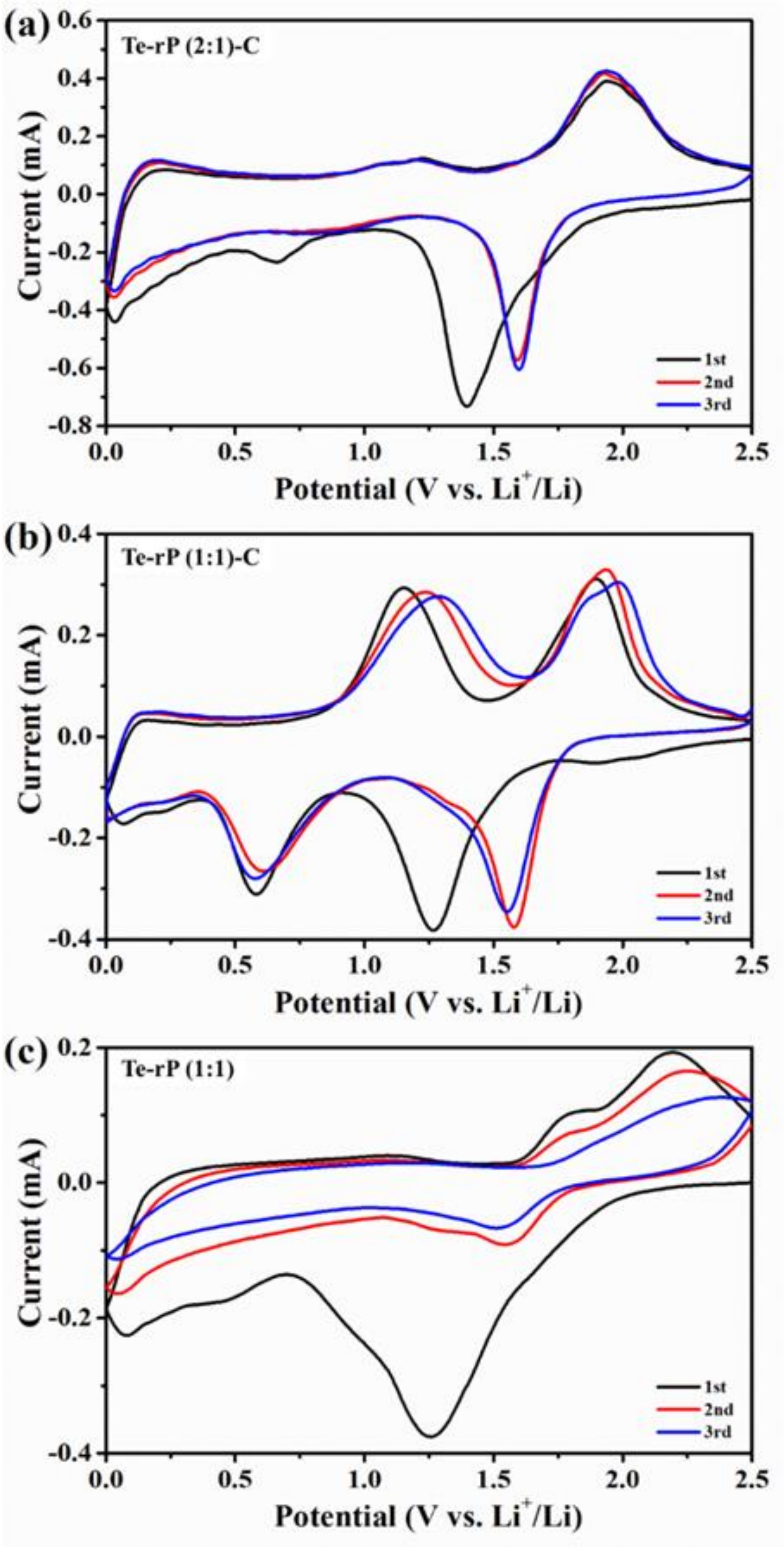

Figure S4. Cyclic voltammetry of (**a**) Te-rP (2:1)-C, (**b**) Te-rP (1:1)-C, and (**c**) Te-rP (1:1) electrodes at 0.1 mV s^{-1}.

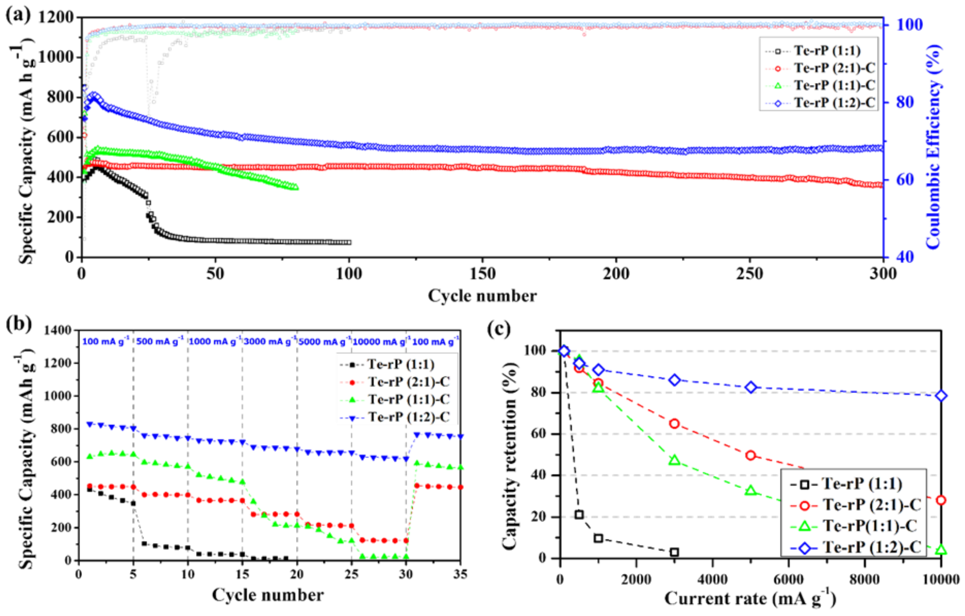

Figure S5. (**a**) Cycling performances of the Te-rP (1:1)-C, Te-rP (2:1)-C, Te-rP (1:1)-C, and Te-rP (1:2)-C without the 5% FEC additive. (**b**) Rate cyclability and (**c**) normalized capacity retention values (percent) of as-prepared electrodes without FEC additive.

Table S1. EIS data of the Te-rP (2:1)-C, Te-rP (1:1)-C, and Te-rP (1:2)-C electrodes.

Sample	R_s(Ω)	R_{SEI}(Ω)	R_{ct}(Ω)	α
Te-rP (2:1)-C	4.66	83.28	27.02	0.5
Te-rP (1:1)-C	1.67	86.29	72.40	0.5
Te-rP (1:2)-C	2.39	82.37	15.77	0.5

Table S2. Comparison of the electrochemical properties of Te, rP-related composite anodes.

Materials	Synthesis method	Electrolyte	ICE	Reversible Capacity	Rate capability	ref
Te-rP (1:2)-C	HEBM	1M $LiPF_6$ in (EC/DEC) with 5% FEC	80%	734 mAh g^{-1} at 100 mA g^{-1} after 300 cycles	600 mAh g^{-1} at 10 A g^{-1}	This study
Te/C	Vacuum-liquid-infusion	1M $LiPF_6$ in (EC/DEC)	27.8%	224 mAh g-1 at 50 mA g-1 after 1000 cycles	55.1 mAh g^{-1} at 4 A g^{-1}	[30]
ZnSe/C	HEBM	1M $LiPF_6$ in (EC/DEC)	76%	705 mAh g^{-1} at 100 mA g^{-1} after 300 cycles	N/A	[53]
CuP_2-Te-C	HEBM	1M $LiPF_6$ in (EC/DEC)	83.6%	532 mAh g^{-1} at 100 mA g^{-1} after 100 cycles	501 mAh g^{-1} at 3 A g^{-1}	[54]
P/AC	Vaporization adsorption	1M $LiPF_6$ in (EC/EMC/DMC)	76.1%	1594 mAh g^{-1} at 140 mA g^{-1} after 50 cycles	730 mAh g^{-1} at 1.43 A g^{-1}	[55]
P-KB	HEBM	1M $LiPF_6$ in (EC/DEC) with 5% FEC	76.4%	1000 mAh g^{-1} at 1000 mA g^{-1} after 300 cycles	600 mAh g^{-1} at 2.4 A g^{-1}	[56]
DWSiNTs	double-walled silicon nanotube	1M $LiPF_6$ in (EC/DEC)	76%	1780 mAh g^{-1} at 400 mA g^{-1} after 200 cycles	540 mAh g^{-1} at 20 C	[57]
GCSi	HEBM	1M $LiPF_6$ in (EC/DEC) with 5% FEC	83%	448 mAh g^{-1} at 500 mA g^{-1} after 100 cycles	N/A	[58]
